# JAK2 V617F as a Marker for Long-Term Disease Progression and Mortality in Polycythemia Vera and its Role in Economic Modeling

**DOI:** 10.36469/jheor.2020.13083

**Published:** 2020-06-04

**Authors:** Jonas Hjelmgren, Kristoffer Nilsson, Gunnar Birgegård

**Affiliations:** 1 The Swedish Institute for Health Economics (IHE), Lund, Sweden; 2 Department of Medical Sciences, Uppsala University, Uppsala, Sweden

**Keywords:** polycythemia vera, JAK2 V617F, economic modeling, validation, disease progression, overall survival

## Abstract

**Background:**

In order to facilitate sound economic evaluations of novel treatments, health-economic models of polycythemia vera (PV) must combine effects on surrogate endpoints in trials with disease progression (DP) and mortality in long-term cohort data.

**Objective:**

We validate an economic model for PV that uses Janus Kinase 2 (JAK2) burden as a surrogate endpoint to predict DP (thrombosis, myelofibrosis, and acute leukemia) and overall survival (OS) based on progression-specific mortality.

**Methods:**

Long-term observational studies that include information about baseline JAK2 burden were identified via PubMed searches and used to validate the model. Kaplan-Meier (KM) OS curves were extracted using a digitizing software. External validity of the model was analyzed by visually comparing OS curves of the model with the KM curves of the included studies, as well as calculating differences in mean OS estimated as area under the curve (AUC).

**Results:**

The model’s predictions of cumulative DP were somewhat lower than the published studies. Over 20 years’ time, our base case model predicted a mean OS for a PV patient (15.0–16.5 years), which was in line with the published studies (15.8–17.5 years). Modeled mean OS was almost two years longer (1.6–1.9 years) for patients with JAK2 <50% than patients with JAK2 ≥50%. Only three long-term observational studies that satisfied the predefined criteria were found and could be used in the validation, but these studies did not capture JAK2 evolution over time. Improved model predictions of DP and mortality based on the longitudinal evolution of JAK2 could be derived from real-world data sources. Such data are currently scarce and future observational studies should be designed to capture the long-term impact of JAK2 on DP and mortality in PV.

**Conclusions:**

Our model, based on JAK2 burden as a marker for DP, generated OS estimations that are in line with results of published data.

## BACKGROUND

Economic evaluations are widely used to inform health technology assessment agencies, such as the National Institute for Health and Clinical Excellence in the United Kingdom, about whether the drug’s incremental benefit (eg, improved overall survival [OS]) stand in proportion to its added costs.^1^ The most widely used decision-making variable in an economic evaluation is the incremental cost-effectiveness ratio (ICER), which is the difference in total cost (TC) of two treatment options (eg, A and B) divided by the difference in total effectiveness (E), and often OS is expressed in life years (LY) (ICER=(TC_A_-TC_B_))/ (LY_A_-LY_B_)). Usually the ICER is expressed as “the cost per LY gained” or as “the cost per quality adjusted life year (QALY) gained.” If the ICER is above a threshold determined by the payer, it will be deemed too expensive and thus should not be funded, whereas the opposite is true for an ICER that falls below the threshold.

Although the decision rules of a cost-effectiveness analysis are easy to apply in theory, the design of an economic evaluation poses a number of challenges. The most prominent one is the collection of data, where the clinical trial is the most essential data source, which ideally would capture all relevant information with regard to long-term improvements in disease progression (DP) and OS. This is however seldom the case, especially in slowly progressing diseases where the majority of the outcomes of interest (for instance, DP or death) manifest after the end of the trial. Even if a longer follow-up was possible, the decision to use a new treatment or not needs to be taken before any observed long-term consequences would be realized. Instead, the primary outcomes in the trial are restricted to surrogate endpoints, which at best are associated with the long-term clinical outcomes of interest. Polycythemia vera (PV) is an example of a slowly progressing disease where trial outcomes mainly consist of effects on surrogate endpoints.

In order to conduct economic evaluations of novel treatments in PV, we have to use health-economic models that combine surrogate endpoints from the trial with long-term observational cohort data of DP and OS. Moreover, to be able to develop such a model, there needs to be an established long-term association between the surrogate endpoint and the clinical outcomes of interest, which in our case are PV-related DP (acute leukemia [AL], myelofibrosis [MF], and thrombosis) and OS.

The primary outcome of most clinical trials of novel treatments targeted at PV is a hematologic response, which primarily includes information about peripheral blood cells (eg, hematocrit <45%), white blood cell and platelet count (WBC <10*10^9^), and bone marrow histology.^2^,^3^ An alternative outcome is a molecular response measured as a Janus Kinase 2 (JAK2) (V617F) mutation allele burden, which is present in 95% to 98% of patients with PV.^4^ – ^8^ Although hematologic and molecular response are both able to differentiate the treatment effects of traditional versus novel treatments,^9^ – ^11^ it has not been verified to what extent these treatments have had any effect on long-term DP or mortality in PV.

We have developed a cost-effectiveness model that uses the JAK2 burden as a surrogate endpoint to predict time to DP (AL, MF, and thrombosis) and death in PV. The objective of this study was to validate the long-term predictions of DP and the OS of our model versus predictions of published real-life observational studies. Possible deviations between model estimates and real-life studies will be explored and discussed. This information is essential to clarify to what extent our model is accurate in predicting DP and OS in PV and to explore whether JAK2 is a sensitive surrogate parameter for predicting DP and OS in PV.

## METHODS

### Model Structure

**Figure 1:**
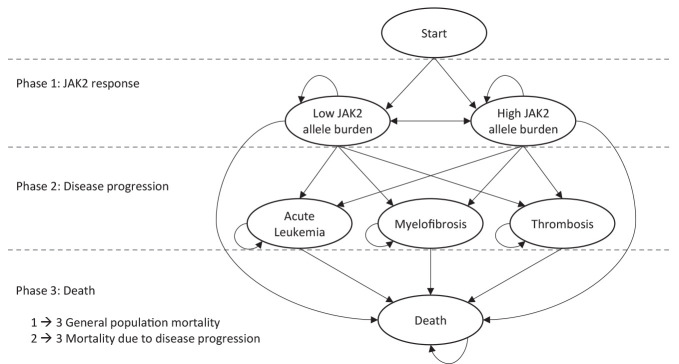
Model Overview Phase 1 consists of the two complication-free states “Low JAK2 allele burden” (<50%) and “High JAK2 allele burden” (>50%). Phase 2 consists of the progression health states “Acute Leukemia” (AL), “Myelofibrosis” (MF), and “Thrombosis”. Phase 3 consists of the final absorbing health state, “Death.” Patients in Phase 1 are assigned general population mortality. After entering Phase 2, patients are assigned complication-specific mortalities. The probabilities of transition from Phase 1 to Phase 2 are dependent on JAK2 burden, except for transition to AL, which is independent of JAK2 burden.

A Markov model aimed at evaluating the cost-effectiveness of novel treatments in PV was developed. An overview of the model structure, with health states (ellipses) and transitions (arrows) between health states, is presented in Figure 1. The patients enter the model in the “Low JAK2 allele burden” (JAK2 burden <50%) or “High JAK2 allele burden” (JAK2 burden ≥50%) health states, and with transitions to the other health states such as AL, MF, thrombosis, or death, each cycle lasts 13 weeks with certain probabilities. Based on the available literature sources, the DP varies depending on JAK2 burden; in the model cycle, probabilities of MF and thrombosis are higher for a JAK2 burden ≥50% versus a JAK2 burden <50%) (Phases 1 to 2) (Table 1). While JAK2 burden affects survival indirectly through the risks of progression, it was assumed to have no direct effect on survival, and patients who have not yet experienced DP were assumed to have general population mortality (Phases 1 to 3). The cycle probabilities to progress to AL, MF, and thrombosis, as well as mortality in these states (Phases 2 to 3), were calculated based on summary estimates (including incidence rates, cumulative incidence, risk ratios, and median OS) from published sources (Table 1).

### JAK2 Burden (Phase 1)

To estimate the longitudinal distribution of patients with JAK2 burdens of <50% and ≥50%, we used a combination of data from the PROUD-PV and the CONTINUATION-PV clinical trials.^12^ The PROUD-PV study was a 12-month Phase 3 open-label, randomized, controlled, parallel-group, non-inferiority study (ropeginterferon alfa-2b vs hydroxyurea [HU]) including both HU-naive and currently treated patients diagnosed with PV. The CONTINUATION-PV was an extension study (to the PROUD-PV study) designed to provide long-term evaluation of ropeginterferon alfa-2b and the best available therapy in patients with PV who received the investigational medicinal product subcutaneously or HU during the PROUD-PV study.

**Figure 2:**
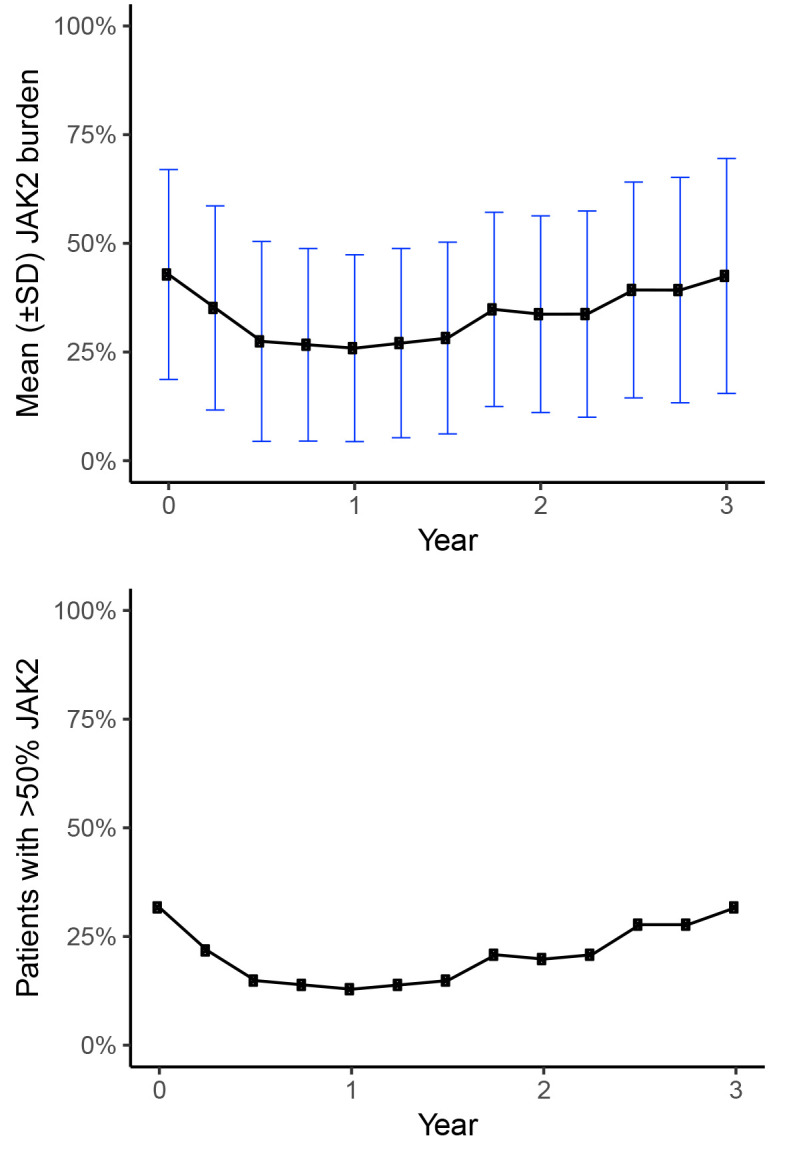
Mean JAK2 burden (HU arm) and modelled proportion of patients with JAK2 burden >50% HU: Hydroxyurea; JAK2: Janus Kinase 2

The model is capable of predicting and evaluating DP and OS for any currently existing PV treatment, but it is also able to assess novel technologies not yet on the market. However, for the purpose of model validation, we need to populate the model with trial data of currently existing treatments since we are then able to compare this with matching real-world evidence. Therefore, we only use the JAK2 data from the HU treatment arm since HU is an established treatment that can be considered a standard of care for patients with PV.^13^,^14^ The combined PROUD-PV and CONTINUATION-PV JAK2 burden results of the 156 weeks of follow-up are presented both in terms of the JAK2 mean value and patients with JAK2 ≥50% for each 13-week cycle (Figure 2). Since the follow-up period of the trial ends at week 156 and we have no knowledge about the future evolution of JAK2, we conservatively assumed that the mean JAK2 burden was at a constant level after week 156.

### Risks of DP (Phase 2)

The risks of DP in the model are shown in Table 1, which indicates the source from which each risk was derived and which risks are active in the base case model. Each risk in the model has been derived from published summary data, and all risks have been transformed to 13-week probabilities. There is evidence of a correlation between the JAK2 burden and the risk of thrombosis and transformation to MF, with especially strong evidence in the latter.^15^ We did not find evidence of a relationship between the JAK2 burden and AL, and therefore both JAK2 groups were assumed to have the same risk of transition to AL.

### Risks of Mortality (Phase 3)

Patients who have not yet experienced DP were assumed to have general population mortality (Phases 1–3), as a direct effect of the JAK2 burden on survival has not yet been reported to our knowledge. The cycle probability of mortality prior to DP is assumed to be the same as for the general population of Sweden (using life tables from Statistics Sweden). Annual rates were converted to 13-week cycle mortality rates. Thirteen-week cycle probabilities of mortality for patients who are in a DP state (Table 2) were derived from observational studies with data on cumulative mortality for PV patients with AL, thrombosis, and MF, respectively.

### Model Validation

Longitudinal mortality (KM analysis) and DP (AL, MF, and thrombosis) data were identified via PubMed searches. The objective was to target longitudinal cohort studies that included information on baseline JAK2 burden and with a follow-up period sufficiently long enough to validate long-term model predictions (>10 years). Details of the literature search are presented in the Supplementary Material. KM survival curves from publications were extracted using the freeware WebPlotDigitizer (version 4.2., San Francisco, CA: Ankit Rohatgi; 2019, https://automeris.io/WebPlotDigitizer), by manually selecting the corners of the graphed lines (in case the breakpoints of the graphs were not clearly visible, the default algorithm was used to depict the graphed line). The extracted data were transferred to the statistical software R (R Core Team (2018), R Foundation for Statistical Computing, Vienna, Austria) where the data points were used to plot the OS curves.

The model validation included visual comparisons of modeled OS curves versus KM survival curves from publications for different time intervals (years 1–10 and 11–20) and comparison of the AUC estimates of mean OS, expressed as LY, of the model and the corresponding estimates of the included publications. The estimates of mean OS are restricted to the investigated time periods (10-year intervals and 20 years in total).

## RESULTS

### Studies Used in the Validation of the Model

Table 2 shows the three selected studies of the literature search used for validation of the model. The included studies were published between 2012 (Malak et al. study) and 2016 (Alvarez-Larrán et al. study), and all studies have maximum follow-up periods extending over a time period of 20 years, which means that they represent a treatment paradigm that goes back to the 1990s. The patients in the Alvarez-Larrán study were stratified into two groups: masked and overt PV. The group with overt PV, according to the WHO definition (n=83), was used in the model validation. The median follow-up period of Malak et al. was twice as long (12 years) as Bai et al. (6 years) and Alvarez-Larrán et al. (6.4 years). Patients appear to be a bit younger in Bai et al. (median age is 54 years) compared to Malak et al. (58 years) and Alvarez-Larrán et al. (64 years). All three studies presented data of hematologic key variables at the baseline that were similar at diagnosis with regard to blood values, age distribution, and number of patients. The JAK2 V617F allele burden >50% was found in 61% of patients in the Bai and Alvarez- Larrán studies compared to 31% in the Malak study. The Malak study had more patients on alkylating agents than Bai (27% vs 4%).

### Model Base Case Results

Table 3 shows the unadjusted base case model results in terms of DP and mean survival over a 20-year time horizon. Starting age was set to 57 and mean JAK2 allele burden at the baseline was 42.8, with a development in JAK2 burden over the first three years as illustrated in Figure 2. The 20 years of cumulative incidence of DP in the simulated PV population was 4%, 12%, and 30% for AL, MF, and thrombosis, respectively. The mean LY over the 20-year period was 16.6 years, while the corresponding figure for the general population was 18.5 years. The model was also run with subgroups with the JAK2 allele burden consistently below or above the 50% threshold. The incidence of MF and thrombosis were higher in the >50% group compared to the <50% group (29.3% vs 2.1% and 37.6% vs 25.8%). The incidence of leukemia was slightly lower in the >50% group than in the <50% group (3.0% vs 4.4%). The risk of leukemia is by construction equivalent between the two groups, but the cumulative incidence differs since the groups have different competing risks (MF and thrombosis) in the model.

### Validation of DP

Cumulative incidence of DP in the model was compared to observed outcomes for each study included in the validation. The model was run with a baseline age and JAK2 burden set to match each of the studies. The model time horizon was set to the same as the time period corresponding to each observed outcome. In the cases where cumulative incidence was not reported for explicitly specified time periods, it was assumed that the time horizon was equal to the median follow-up of the validation study.

Overall, the cumulative incidences observed in the model were lower than in the studies included in the validation (Table 4). The cumulative median 12 years of AL incidence in Malak et al. was 7.9 times higher than the prediction of the model (22% vs 2.8%), whereas incidence of MF and thrombosis were 2.7 (21% vs 7.9%) and 2.0 times higher (42% vs 21%), respectively. The cohort in Bai et al. study had six years of median follow-up but reported relatively high numbers of complications. Cumulative incidence of AL was 3.7 times higher compared to the model (5.5% vs 1.5%) whereas MF and thrombosis were about 3.0 (23% vs 7.6%) and 3.1 (44% vs 14%) times higher, respectively. When compared to the cohort in Alvarez-Larrán et al., the cumulative incidences of the model were close, with minor differences in AL (3.6% vs 2.3%) and thrombosis (22.5% vs 21%) and a small difference in MF (14.0% vs 11.3%).

### Validation of OS and KM Curves

**Figure 3:**
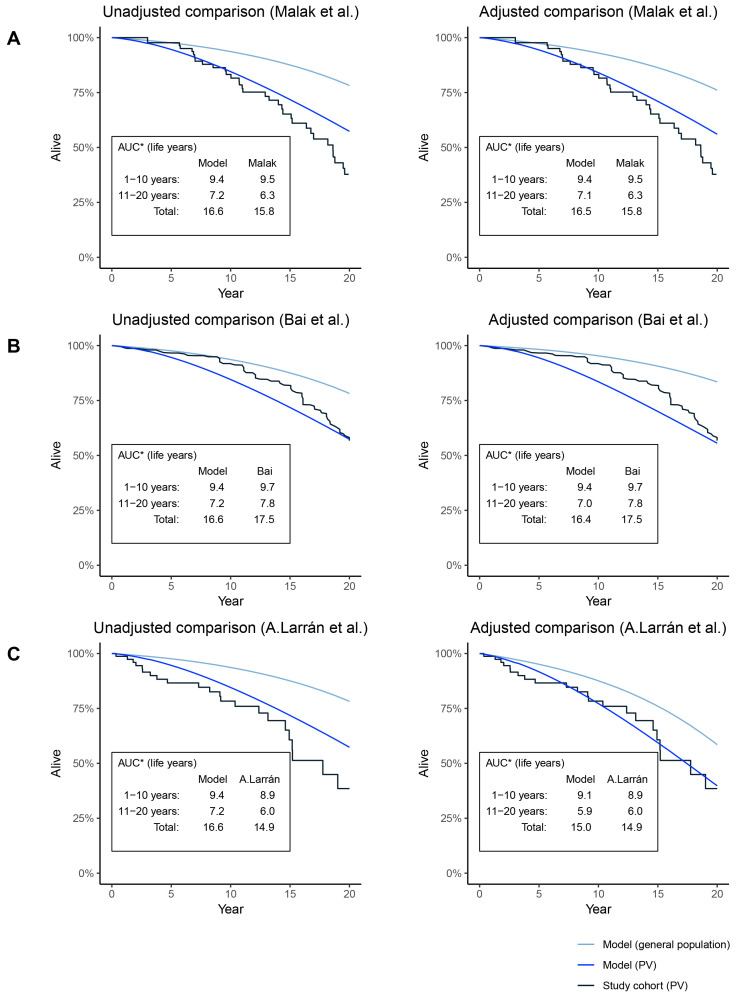
Comparison of Survival Curves Generated by Model and KM Curves of Real-Life Studies *AUC calculated as: $\sum_{t=0}^{T-1}S(t)\Delta t$, *S*, survival.

Figure 3A–C illustrates the KM curves of the cohorts from the included studies and the model OS curves, using both unadjusted and adjusted models. In the adjusted models, mean age and baseline mean JAK2 have been equalized between the model and the studies.

The comparison between the adjusted model and the cohort in Malak et al. (Figure 3A) indicates a good visual fit for the first 10 years (AUC was 9.4 and 9.5 LY for the model and study cohort, respectively), whereas there is some divergence after year 10 up to year 20 (model: 7.1 LY; study cohort: 6.3 LY). In this case, the difference in survival between the adjusted and unadjusted model was very limited.

When comparing the model with the cohort in Bai et al. (Figure 3B), there was some divergence starting from the middle of the first 10 years (adjusted model: 9.4 LY; study cohort: 9.7 LY). After about 15 years, the mortality in the study cohort seems worse than the model, which leads to a convergence of the survival between year 11 and 20 (adjusted model: 7.0 LY, study cohort: 7.8 LY). The adjustments of JAK2 and age in the model had an insignificant impact on the results. The baseline age was adjusted downwards and the mean JAK2 burden was adjusted upwards, which explains why the survival of the overall PV population is slightly worse compared to the unadjusted model, while at the same time, the general population survival was higher in the adjusted model.

The unadjusted model had higher survival over 20 years than the cohort in Alvarez-Larrán et al. (Figure 3C), which was expected due to the age difference (57 and 64 years in model and study, respectively). Adjustments led to a good overall fit and the AUC was close to equal for years 1–10 and years 11–20 (adjusted model: 9.1 and 5.9 LY; study cohort: 8.9 and 6.0 LY).

### Impact on OS of Using Different Sets of Risks of Progression and Mortality

Sensitivity analyses were performed for each of the three validation studies by setting baseline characteristics according to the validation study and applying different sets of risk levels (Table 1) for progression (AL, thrombosis, and MF) and mortality. Using different combinations of the available risk levels in the model resulted in a range of estimated mean OS for each validation study. In the comparison between the model and the cohort in Malak et al., the estimated 20-year survival ranged between 15.8 and 17.1 LY (16.5 LY in the base case), which means that the lower bound of the range just reached the estimate from the study cohort (15.8 LY). In the comparison between the model and the cohort in Bai et al., the estimated 20-year survival ranged between 15.4 and 17.3 LY (16.4 LY in the base case). In this case, the upper bound of the range was still slightly below the study cohort (17.5 LY). In the comparison between the model and the cohort in Alvarez-Larrán et al., the estimated 20-year survival ranged between 14.2 and 15.8 (15.0 in base case), meaning that the range of estimated survival overlapped that of the study cohort (14.9 LY).

### OS for Patients JAK2 <50% and JAK2 ≥50%

**Figure 4:**
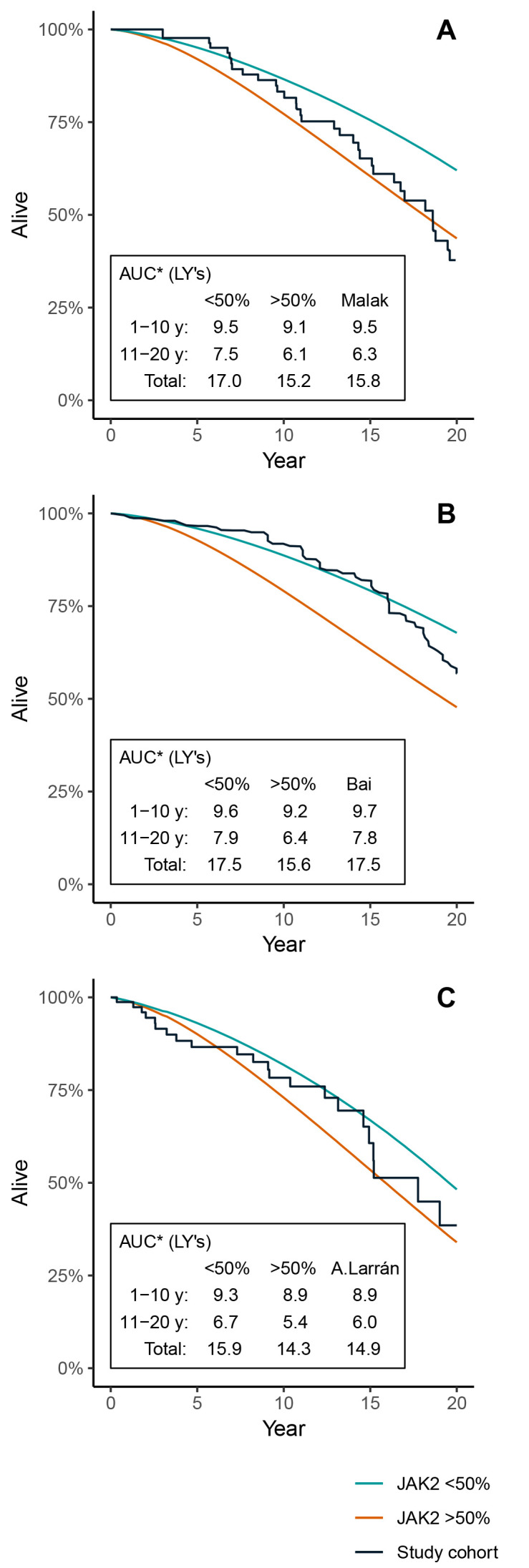
KM Curves of Real-Life Studies and Modeled Survival Curves Depending on Patients’ JAK2 Burden *AUC calculated as: $\sum_{t=0}^{T-1}S(t)\Delta t$, *S,* survival.

To further illustrate what potential impact the JAK2 burden has in the model, the model was run with subgroups with the JAK2 allele burden consistently below or above the 50% threshold, with a starting age set to match the validation cohorts. Figure 4A–C illustrates the KM curve of each validation cohort, compared to the model OS curves when keeping all patients at either >50% or <50% JAK2 burden throughout the simulation. The validation cohorts from Malak et al. (Figure 4A) and Alvarez-Larrán et al. (Figure 4C) seem to have survival curves located within the modeled curves of patients with a high and low JAK2 burden, whereas the cohort from Bai et al. (Figure 4B) is closer to the model scenario with low JAK2. There seems to be a general tendency that the observed survival curves of the validation cohorts are skewed in the sense that they are initially flat and then, after 10 to 15 years, begin to steepen.

## DISCUSSION

Economic models help health technology assessment organizations and payers make sound judgments on the potential health benefits that may be manifested far beyond the time horizon of the clinical trial and put these into the context of the high expenses that are payed prior to the realization of the benefits. The accuracy of the models in predicting DP and OS is fundamental in order to properly assess to what extent a PV drug brings value to patients and society. Trust and confidence are critical to the success of the economic models, and validation of how well the model reflects “reality” is a key component of the model development process.^16^ The question is then whether payers could put trust and confidence in a model that uses a surrogate endpoint such as JAK2 to predict DP and survival of PV?

We developed an economic model for PV that combines data from multiple sources such as clinical trials and long-term observational cohort studies.^12^,^15^,^17^–^30^ The objective of this study was to validate the model that uses JAK2 burden as a surrogate endpoint to predict DP and OS based on progression-specific mortality.

The results of the validation indicated a good correspondence between the model and the published observational studies available for comparisons. The mean OS in the published observational studies^19^,^31^,^32^ that was included in the validation of the model was 14.9 to 17.5 years over a 20-year time horizon. When we adjusted our model’s baseline age and baseline JAK2 burden to match the studies’ variables, our model predicted a mean OS that ranged between 15.0 to 16.5 years. When we combined risk levels to establish a range of possible OS levels, our model provided an OS range of 14.2 to 17.3 LY depending on age and baseline JAK2. Only the study by Bai et al^19^ had an OS (17.5 LY) that slightly fell outside of the modeled OS range.

The cumulative incidence of MF, leukemia, and thrombosis were considerably lower in the model for the average population than in the studies, except for the Alvarez-Larrán study^32^ where the incidences were relatively similar. For separate model simulations of JAK2 subgroups, the incidences of MF and thrombosis reached considerably higher levels for patients with JAK2 >50%, which might imply that possible differences in JAK2 levels across different studies could explain differences in incidence of MF and thrombosis.

Differences in predictions of the model and the outcomes in the observational studies could partly be contextual in the sense that our model was based on data from multiple sources where the patient populations differed from the populations in the studies that were included in the validation. For instance, the study by Bai et al^19^ included only Chinese patients, who had a higher incidence of MF transformation than western cohorts. The treatment pattern in the Bai et al study also differed in the sense that it included considerably more patients treated with interferon-α alone, which has been shown to have a positive effect on OS.^33^ The patients in the Malak study^31^ had a longer observation time and treatment pattern with increased leukemia transformation risk, which may account for a higher leukemia incidence and higher mortality. Also, the Malak study included only patients with familial myeloproliferative neoplasms, which may have different characteristics than sporadic myeloproliferative neoplasms.

Another explanation for the differences could simply be statistical uncertainty as the studies included in the validation were rather small with sample sizes ranging from 83 to 272 patients. Although we encountered observational studies with larger cohorts of patients with PV in the literature search, such studies did not meet the inclusion criteria of a long follow-up and the data on JAK2 burden and mortality. Limited data are a general problem for orphan diseases such as PV and add to the complexity of making sound economic assessments of technologies aimed at treating these diseases.^34^ One way to reduce this uncertainty in modeling is to combine data from good quality clinical trials, including appropriate and relevant markers of DP and OS, with long-term longitudinal observational registry data. Such registries are to some extent already available, and publications have presented results of associations between hematocrit levels (eg, Crisa et al., 2010),^35^ white blood cell counts (eg, Tefferi et al., 2013),^36^ and OS in PV patients. Although these studies included considerably more patients (up to 1545) than the studies that were used to validate the model, we were not able to use them since no JAK2 data were available in these publications.

One limitation with the published data we had at hand was that we were not able to identify the evolution of JAK2 over time and we were therefore not able to adjust our model based on these potential variations. Instead, the same three-year evolution of JAK2 as observed in the HU treatment arm in the PROUD-PV and CONTINUATION-PV was assumed to apply, as we only adjusted the baseline JAK2 values to match the observational studies. When we compared the OS estimates of our model with the published studies, we were not able to find a consistent pattern of model versus the studies with respect to the time horizon; in one case, the fit was better during the first 10-year period compared to the second 10-year period, and in another case, the model showed lower survival than the study in the first 10-year period and then higher survival than the study in the second 10-year period. However, when we simulated OS for patients with JAK2 that is <50% and JAK2 that is ≥50 separately, we noticed that predictions based on JAK2 that is <50% generally fit the OS of the studies better up to year 10, whereas the OS predictions of JAK2 that is ≥50 had a better fit with the studies after year 10. The results might suggest that a change of JAK2 status over time influences OS for patients with PV. The clinical trial data that were used in the model did not provide observations of JAK2 burden after year three. Therefore, the level of JAK2 burden in our model was kept constant after year three and did not capture the potential longitudinal effect a higher JAK2 burden may have on the rate of DP and mortality.

Future observational studies should be designed to capture the effect of JAK2 burden and its long-term impact on DP and mortality. It is especially important to investigate the impact various treatments might have on JAK2 evolution over time, which might provide valuable information for development of existing treatment guidelines. Furthermore, a more granular cutoff than JAK2 that is >50% might be beneficial to provide guidance in clinical practice and to identify when a drug provides incremental benefit compared to other alternatives.

Another limitation with the observational studies was the lack of reported numerical estimations of OS over time. To obtain OS estimates for different time points, we had to extract KM data using a digitizer software. We thus had to rely on the KM data plots being correct and reflecting the actual study result. The digitizer tool we used has generated data into several other published modeling studies,^37^–^40^ and we feel confident that the results that formed the basis of our model validation were reasonably reliable.

One might argue that our validation only included three studies and therefore lacks enough power to evaluate whether our model provides sound estimates of DP and OS. However, the results of our model validation clearly show that comparisons of study results are complex and are not just about the number of studies included. For instance, we would believe that high incidences of MF and thrombosis in a PV population would result in shorter survival. However, the Bai et al study had both higher rates of MF and thrombosis and also had longer OS than the other two studies and our model. This is a relationship that is difficult to explain a priori, and in order to validate models in the best possible way, we would need to extract information from publications that might be impossible to obtain.

Apart from the methodologic aspects of using JAK2 as a surrogate endpoint, one may ask why we used this endpoint? We see two important reasons for establishing a model that associates molecular response expressed as JAK2 burden to DP and OS. Firstly, molecular response is not included as part of the definition of response in PV^2^ and seems to have a “lower” status compared to the established hematologic response definition, despite strong evidence for a correlation between JAK2 burden and transformation to MF.^15^ Secondly, novel agents in use today have been able to reduce allele burden of the JAK2 V617F mutation in PV, but there is poor knowledge about the potential long-term consequences of keeping control of JAK2 burden.^9^–^11^ Separate simulations with our model for patients with JAK2 that is ≤50% and >50% indicate that individuals in the former group had about two years longer survival. Both of these previously stated arguments could be motivation for updating the existing treatment guidelines for PV.

## CONCLUSIONS

Our results indicate that it is possible to use JAK2 as a marker for predicting DP and ultimately OS in PV. More powerful long-term observational studies are however needed to provide more robust long-term predictions of absolute magnitude. The results of model simulations, which showed that individuals with a lower JAK2 burden had longer survival, suggest that the JAK2 burden should be followed in clinical practice to monitor the treatment success of novel interventions.

## Supplementary Information



## Figures and Tables

**Table 1:** Risk of Disease Progression and Mortality in Disease Progression for Data Used in BC Setting and Scenario Analysis

Source for Deriving Risks	Used in BC (Yes/No)	Probability per 13-Week Cycle	
		JAK2 <50%	JAK2 ≥50%
**Risk of Disease Progression per 13-Week Cycle**			
Progression to Thrombosis			
Alvarez-Larrán et al. (2014)^15^	Yes	0.004	0.009
Vannuchi et al. (2007)^17^	No	0.005	0.016
Progression to Leukemia			
Finazzi et al. (2005)^18^	Yes	0.00073	0.00073
Tefferi et al. (2013)^36^	No	0.00058	0.00058
Progression to Myelofibrosis			
Alvarez-Larrán et al. (2014)^15^	Yes	0.0003	0.007
Bai et al. (2015)^19^	No	0.0003	0.002
Passamonti et al. (2010)^20^	No	0.0006	0.007
**Risk of Mortality per 13-Week Cycle**			
Mortality in Thrombosis			
Marchioli et al. (2005)^21^	Yes	0.018	0.018
Di Veroli et al. (2018)^22^	No	0.012	0.012
Mortality in Leukemia			
Chihara et al. (2016)^23^	Yes	0.26	0.26
Juliusson et al. (2009)^24^	No	0.16	0.16
Kennedy et al. (2013)^25^	No	0.27	0.27
Lancman et al. (2018)^26^	No	0.30	0.30
Passamonti et al. (2005)^27^	No	0.51	0.51
Mortality in Myelofibrosis			
Passamonti et al. (2017)^28^	Yes	0.021	0.021
Cervantes et al. (2009)^29^	No	0.029	0.029
Masarova et al. (2017)^30^	No	0.042	0.042

Abbreviations: BC, base case; JAK2, Janus Kinase 2.

**Table 2:** Patient Population Characteristics of Validation Studies and Trial

	Malak et al. 2012^31^	Bai et al. 2015^19^	Alvarez-Larrán et al. 2016^32^	PROUD-PV/CONTINUATION-PV (HU arm)
**Treatment Context**				
Country	France	China	Spain	
Type of Study	Retrospective analysis of medical records.	Retrospective cohort identified in data base.	Retrospective cohort study.	
**Key Study Parameters**				
Sample Size PV Population	93	272	83	127/76
Age at Diagnosis (Median)	58	54	64	58/58
Median Follow-Up (Years)	12	6	6	
WBC Count (× 10^9^/L)	12	14	11	12/12
Hemoglobin Level (g/L)	187	200	192	NA/157
Platelet Count (× 10^9^/L)	529	420	424	547/512
Hematocrit (%)	56	NA	60	49/50
JAK2 (% Above 50% or Median Allele Burden)	31% of patients >50%	61% of patients >50%^b^	Median allele burden 64%	Median allele burden 43%/43%
**Cytoreductive Drugs**				
No Cytoreductive Drug (%)	3	7	NA	NA
IFNα Alone (%)	NA	34	NA	NA/3^a^
Hydroxyurea Alone (%)	46	55	NA	100/97^a^
Alkylating Agent (%)	27	4	NA	NA
Combination (%)	24	NA	NA	NA

Abbreviations: PV, polycythemia vera; HU, Hydroxyurea; WBC, White blood cell; JAK2, Janus Kinase 2; IFNα, Interferon alfa.

a At month 36.

b JAK2 was assessed in 90 of 272 patients.

**Table 3:** Model Simulation Results Using Unadjusted Baseline Values for Age and JAK2

Model Base Case Results for Time Horizon of 20 Years	Model Simulation Based on PV Population with HU Treatment in PROUD/ COUTINUATION	General Population	Model Simulations for JAK2 Subgroups	
			JAK2 <50%	JAK2 ≥50%
**Disease Progression (%)**				
Acute leukemia	3.98	-	4.42	3.04
Myelofibrosis	11.88	-	2.06	29.30
Thrombosis	30.40	-	25.84	37.63
Overall Survival (LY)	16.6	18.5	17.1	15.3

Abbreviations: PV, polycythemia vera; HU, Hydroxyurea; JAK2, Janus Kinase 2.

**Table 4:** Cumulative Incidence in Percent and Annual Rate of Disease Progression, Model, and Study Cohorts

		Malak et al. (2012)^31^ (12-Year Follow-Up)		Bai et al. (2015)^19^ (6-Year Follow-Up)		Alvarez-Larrán et al. (2016)^32^ (10-Year Follow-Up)	
	Cumulative Incidence (%)	Total (12 Years)	Annual Rate	Total (6 Years)	Annual Rate	Total (10 Years)	Annual Rate
**Validation Study Cohort**	Leukemia	22.0	2.1	5.5	0.9	3.6	0.4
	Myelofibrosis	21.0	2.0	23.2	4.4	14.0	1.5
	Thrombosis	42.0	4.5	44.1	9.7	22.5	2.5
**Model (Adjusted)**	Leukemia	2.8	0.2	1.5	0.3	2.3	0.2
	Myelofibrosis	7.9	0.7	7.6	1.3	11.3	1.2
	Thrombosis	21.1	2.0	13.9	2.5	20.5	2.3

## References

[1] Brazier J, Ratcliffe J, Saloman J, Tsuchiya A (2016). Measuring and Valuing Health Benefits for Economic Evaluation.

[2] Barosi G, Mesa R, Finazzi G (2013). Revised response criteria for polycythemia vera and essential thrombocythemia: an ELN and IWG-MRT consensus project. Blood.

[3] Tefferi A, Cervantes F, Mesa R (2013). Revised response criteria for myelofibrosis: International Working Group-Myeloproliferative Neoplasms Research and Treatment (IWG-MRT) and European LeukemiaNet (ELN) consensus report. Blood.

[4] James C, Ugo V, Le Couedic JP (2005). A unique clonal JAK2 mutation leading to constitutive signalling causes Polycythaemia vera. Nature.

[5] Kralovics R, Teo SS, Buser AS (2005). Altered gene expression in myeloproliferative disorders correlates with activation of signaling by the V617F mutation of JAK2. Blood.

[6] Levine RL, Wadleigh M, Cools J (2005). Activating mutation in the tyrosine kinase JAK2 in Polycythemia vera, essential thrombocythemia, and myeloid metaplasia with myelofibrosis. Cancer Cell.

[7] Baxter EJ, Scott LM, Campbell PJ (2005). Acquired mutation of the tyrosine kinase JAK2 in human myeloproliferative disorders. Lancet (London, England).

[8] Brecqueville M, Rey J, Bertucci F (2012). Mutation analysis of ASXL1, CBL, DNMT3A, IDH1, IDH2, JAK2, MPL, NF1, SF3B1, SUZ12, and TET2 in myeloproliferative neoplasms. Genes, Chromosomes Cancer.

[9] Vannucchi AM, Verstovsek S, Guglielmelli P (2017). Ruxolitinib reduces JAK2 p.V617F allele burden in patients with Polycythemia vera enrolled in the RESPONSE study. Ann Hematol.

[10] Bose P, Verstovsek S (2019). Updates in the management of Polycythemia vera and essential thrombocythemia. Ther Adv Hematol.

[11] Kiladjian JJ, Cassinat B, Chevret S (2008). Pegylated interferon-alfa-2a induces complete hematologic and molecular responses with low toxicity in Polycythemia vera. Blood.

[12] Gisslinger H, Klade C, Georgiev P (2020). Ropeginterferon alfa-2b versus standard therapy for Polycythaemia vera (PROUD-PV and CONTINUATION-PV): a randomised, non-inferiority, phase 3 trial and its extension study. The Lancet Haematology.

[13] Tefferi A, Barbui T (2019). Polycythemia vera and essential thrombocythemia: 2019 update on diagnosis, risk-stratification and management. Am J Hematol.

[14] Barbui T, Barosi G, Birgegard G (2011). Philadelphia-negative classical myeloproliferative neoplasms: critical concepts and management recommendations from European LeukemiaNet. J Clin Oncol.

[15] Alvarez-Larrán A, Bellosillo B, Pereira A (2014). JAK2V617F monitoring in polycythemia vera and essential thrombocythemia: clinical usefulness for predicting myelofibrotic transformation and thrombotic events. Am J Hematol.

[16] Eddy DM, Hollingworth W, Caro JJ (2012). Model transparency and validation: a report of the ISPOR-SMDM Modeling Good Research Practices Task Force—7. Value in Health: The Journal of the International Society for Pharmacoeconomics and Outcomes Research.

[17] Vannucchi AM, Antonioli E, Guglielmelli P (2007). Prospective identification of high-risk polycythemia vera patients based on JAK2(V617F) allele burden. Leukemia.

[18] Finazzi G, Caruso V, Marchioli R (2005). Acute leukemia in polycythemia vera: an analysis of 1638 patients enrolled in a prospective observational study. Blood.

[19] Bai J, Ai L, Zhang L, Yang FC, Zhou Y, Xue Y (2015). Incidence and risk factors for myelofibrotic transformation among 272 Chinese patients with JAK2-mutated polycythemia vera. Am J Hematol.

[20] Passamonti F, Rumi E, Pietra D (2010). A prospective study of 338 patients with polycythemia vera: the impact of JAK2 (V617F) allele burden and leukocytosis on fibrotic or leukemic disease transformation and vascular complications. Leukemia.

[21] Marchioli R, Finazzi G, Landolfi R (2005). Vascular and neoplastic risk in a large cohort of patients with polycythemia vera. J Clin Oncol.

[22] Di Veroli A, Buccisano F, Andriani A (2018). Prognostic factors for thrombosis-free survival and overall survival in polycythemia vera: a retrospective analysis of 623 PTS with long follow-up. Leuk Res.

[23] Chihara DKH, Newberry KJ (2016). Survival outcome of patients with acute myeloid leukemia transformed from myeloproliferative neoplasms. Blood Rev.

[24] Juliusson G, Antunovic P, Derolf A (2009). Age and acute myeloid leukemia: real world data on decision to treat and outcomes from the Swedish Acute Leukemia Registry. Blood.

[25] Kennedy JA, Atenafu EG, Messner HA (2013). Treatment outcomes following leukemic transformation in Philadelphia-negative myeloproliferative neoplasms. Blood.

[26] Lancman G, Brunner A, Hoffman R, Mascarenhas J, Hobbs G (2018). Outcomes and predictors of survival in blast phase myeloproliferative neoplasms. Leuk Res.

[27] Passamonti F, Rumi E, Arcaini L (2005). Leukemic transformation of polycythemia vera: a single center study of 23 patients. Cancer.

[28] Passamonti F, Giorgino T (2017). A clinical-molecular prognostic model to predict survival in patients with post polycythemia vera and post essential thrombocythemia myelofibrosis. Leukemia.

[29] Cervantes F, Dupriez B, Pereira A (2009). New prognostic scoring system for primary myelofibrosis based on a study of the International Working Group for Myelofibrosis Research and Treatment. Blood.

[30] Masarova L, Bose P, Daver N (2017). Patients with post-essential thrombocythemia and post-polycythemia vera differ from patients with primary myelofibrosis. Leuk Res.

[31] Malak S, Labopin M, Saint-Martin C, Bellanne-Chantelot C, Najman A (2012). Long term follow up of 93 families with myeloproliferative neoplasms: life expectancy and implications of JAK2V617F in the occurrence of complications. Blood Cells, Mol, Dis.

[32] Alvarez-Larrán A, Angona A, Ancochea A (2016). Masked Polycythaemia vera: presenting features, response to treatment and clinical outcomes. Eur J Haematol.

[33] Crisa E, Cerrano M (2017). Can pegylated interferon improve the outcome of polycythemia vera patients?. J Hematol Oncol.

[34] Pearson I, Rothwell B, Olaye A, Knight C (2018). Economic modeling considerations for rare diseases. Value in Health: The Journal of the International Society for Pharmacoeconomics and Outcomes Research.

[35] Crisa E, Venturino E, Passera R (2010). A retrospective study on 226 polycythemia vera patients: impact of median hematocrit value on clinical outcomes and survival improvement with anti-thrombotic prophylaxis and non-alkylating drugs. Ann Hematol.

[36] Tefferi A, Rumi E, Finazzi G (2013). Survival and prognosis among 1545 patients with contemporary polycythemia vera: an international study. Leukemia.

[37] Bagepally BS, Gurav YK, Anothaisintawee T, Youngkong S, Chaikledkaew U, Thakkinstian A (2019). Cost utility of sodium-glucose cotransporter 2 inhibitors in the treatment of metformin monotherapy failed type 2 diabetes patients: a systematic review and meta-analysis. Value in Health: The Journal of the International Society for Pharmacoeconomics and Outcomes Research.

[38] Coyle D, Villeneuve PJA (2019). Economic evaluation of azacitidine in elderly patients with acute myeloid leukemia with high blast counts. *PharmacoEconomics - Open*.

[39] Capri S, Barbieri M, de Waure C, Boccalini S (2018). Cost-effectiveness analysis of different seasonal influenza vaccines in the elderly Italian population. Hum Vaccines Immunother.

[40] Diaby V, Adunlin G, Ali AA (2016). Cost-effectiveness analysis of 1st through 3rd line sequential targeted therapy in HER2-positive metastatic breast cancer in the United States. Breast Cancer Res Treat.

