# Supplementary material for: JAK2 V617F as a Marker for Long-Term Disease Progression and Mortality in Polycythemia Vera and its Role in Economic Modeling
Source: J Health Econ Outcomes Res. 2020 Jun 4;7(1):61–70. doi: 10.36469/jheor.2020.13083 (PMC7343343; doi:10.36469/jheor.2020.13083)
Supplement: Supplementary file 1 [file jheor-7-1-13083-s01.pdf]

Journal of Health Economics and Outcomes Research

Supplementary Online Material

Hjelmgren J, Nilsson K, Birgegård G. JAK2 V617F as a marker for long-term disease progression and mortality in polycythemia vera and its role in economic modeling. *JHEOR*. 2020;7(1):61-70.
doi: 10.36469/jheor.2020.13083

LITERATURE SEARCH

To restrict the number of potential articles we focused on those that reported both mortality and disease progression. Studies were identified via PubMed searches. Combined search terms included "polycythemia vera", "cohort", "long-term", "progression" and "overall survival", as well as MeSH terms "Polycythemia Vera", "Survival Analysis" and ("Janus Kinase 2" or "Follow-Up Studies"). A total 84 studies were found of which 5 were duplicates and removed before screening. Of the 79 studies screened 37 were reviews (11), non-English (2), small sample (1) or not PV (23) and was therefore excluded and not assessed. 42 abstracts were assessed of which 5 were excluded due to small sample size, and 34 due to lack of PV related outcomes of interest for validation (mortality, disease progression and JAK2 burden). Three studies included sufficient information with regard to long-term mortality (Kaplan-Maier analysis), base-line age of the population and presence of JAK2 baseline burden and were therefore included in the analysis. One of the included studies was also previously used when calculating risks of progression in the model. This study is included in the analysis and viewed as "partially independent" following the definitions of ISPOR-SMDM 1.

REFERENCES

1. Eddy DM, Hollingworth W, Caro JJ, et al. Model transparency and validation: a report of the ISPOR-SMDM Modeling Good Research Practices Task Force—7. *Value in Health: The Journal of the International Society for Pharmacoeconomics and Outcomes Research*. 2012;15(6):843–850.

This is an open-access article distributed under the terms of the Creative Commons Attribution 4.0 International License (CCBY-4.0). View this license's legal deed at http://creativecommons.org/licenses/by/4.0 and legal code at http://creativecommons.org/licenses/by/4.0/legalcode for more information.
